# Gene Flow between Sympatric Life History Forms of *Oncorhynchus mykiss* Located above and below Migratory Barriers

**DOI:** 10.1371/journal.pone.0079931

**Published:** 2013-11-05

**Authors:** Donald M. Van Doornik, Barry A. Berejikian, Lance A. Campbell

**Affiliations:** 1 Northwest Fisheries Science Center, National Marine Fisheries Service, National Oceanic and Atmospheric Administration, Manchester, Washington, United States of America; 2 Washington Department of Fish and Wildlife, Olympia, Washington, United States of America; Institut National de la Recherche Agronomique (INRA), France

## Abstract

*Oncorhynchus mykiss* have a diverse array of life history types, and understanding the relationship among types is important for management of the species. Patterns of gene flow between sympatric freshwater resident *O. mykiss*, commonly known as rainbow trout, and anadromous *O. mykiss*, commonly known as steelhead, populations are complex and poorly understood. In this study, we attempt to determine the occurrence and pathways of gene flow and the degree of genetic similarity between sympatric resident and anadromous *O. mykiss* in three river systems, and investigate whether resident *O. mykiss* are producing anadromous offspring in these rivers, two of which have complete barriers to upstream migration. We found that the population structure of the *O. mykiss* in these rivers appears to be influenced more by the presence of a barrier to upstream migration than by life history type. The sex ratio of resident *O. mykiss* located above a barrier, and smolts captured in screw traps was significantly skewed in favor of females, whereas the reverse was true below the barriers, suggesting that male resident *O. mykiss* readily migrate downstream over the barrier, and that precocious male maturation may be occurring in the anadromous populations. Through paternity analyses, we also provide direct confirmation that resident *O. mykiss* can produce offspring that become anadromous. Most (89%) of the resident *O. mykiss* that produced anadromous offspring were males. Our results add to the growing body of evidence that shows that gene flow does readily occur between sympatric resident and anadromous *O. mykiss* life history types, and indicates that resident *O. mykiss* populations may be a potential repository of genes for the anadromous life history type.

## Introduction

Salmonids show extensive within-species life history variation in a variety of traits, such as age and size at maturity, seasonal spawn timing, and migration and mating behavior. Such diversity is believed to increase resilience to unfavorable environmental variables, and buffers population fluctuations over time [1]. Many salmonid species have both an anadromous life history type, which migrates from freshwater to saltwater, and then returns to freshwater to spawn, and a resident life history type, which remains in fresh water for its entire life. Anadromy allows for individual fish to take advantage of the greater productivity in the ocean compared to freshwater [2], resulting in greater growth opportunity that should then lead to greater reproductive output [3]. It comes with a cost, however, as anadromous individuals may experience greater predation and greater physiological stress and energy expenditure that is involved with migrating between fresh and salt water. The propensity to migrate to salt water may be dependent on numerous factors such as population density, condition of individuals, and sex [3]. The persistence and frequency of both anadromous and resident types, often in sympatry, suggests that neither one has a consistently greater fitness. This could be due to fluctuations in the variables that determine which type is favored, resulting in frequent changes over time in which life history type is favored [4].

The salmonid species *Oncorhynchus mykiss* can occur as either a freshwater resident type, commonly known as rainbow trout, or an anadromous type, known as steelhead, both of which frequently occur in sympatry [5]. Resident *O. mykiss* spend their entire lives in freshwater streams or lakes, whereas anadromous *O. mykiss* migrate from their place of birth in freshwater to the marine environment where they live for a variable number of years before maturing and returning to freshwater (usually the same stream in which they were born) to spawn. The relationship between resident *O. mykiss* and anadromous *O. mykiss* populations where they occur in the same river, particularly in regard to gene flow between the two types, is important to discern for proper management of the populations. If there is significant gene flow between the two life history types in the same river, then they both may need to be included in the same conservation unit for purposes of managing that population [6].

Patterns of gene flow between resident and anadromous *O. mykiss* populations are no doubt complex and may differ among river systems. A few locations have shown significant genetic differences between sympatric resident and anadromous *O. mykiss* populations [7,8], but most have not [8-14]. This implies that significant gene flow can occur between sympatric resident and anadromous *O. mykiss* populations, despite the fact that the propensity to migrate to saltwater appears to be under genetic control [15-17] and is strongly, but not completely, influenced by parental life history type [18]. Otolith microchemistry analysis has confirmed that female resident *O. mykiss* can produce anadromous offspring, and that female anadromous *O. mykiss* can produce resident offspring [7,19-21]. In addition, the propensity to smolt and tolerate saltwater can be highly heritable in *O. mykiss* [15]. However, no direct links have been shown between paternal life history and offspring life history in natural populations. Speculation and indirect evidence suggests that resident *O. mykiss* males spawn readily with anadromous *O. mykiss* females [5,22-25]. However, such matings have not been confirmed empirically. In a small southeast Alaska population, all possible mating combinations between resident *O. mykiss* and anadromous *O. mykiss* produced both life history types, but the resident *O. mykiss* were from a landlocked population that had originally been derived from *O. mykiss* residing in the below-barrier, anadromous portion of the stream, and all offspring were raised in a captive rearing environment [15]. The occurrence and frequency of the paternal contribution to “life history switching” (one form producing the other) in natural populations of *O. mykiss* are unknown.

The possibility that gene flow occurs between sympatric resident and anadromous *O. mykiss* populations leads to several considerations. Resident *O. mykiss* may provide a reservoir of genetic material for the anadromous *O. mykiss* population if they are similar and interbreed regularly without compromising fitness. Such a situation would be critically important for anadromous *O. mykiss* populations for which there are conservation concerns. Resident *O. mykiss* could also help to maintain a larger effective population size in an *O. mykiss* population [26], another important consideration for populations with conservation concerns. On the other hand, introgression of resident *O. mykiss* genes could have a detrimental effect on anadromous *O. mykiss* populations by reducing the proportion of individuals that migrate to sea and their fitness in the marine environment [27], to the extent there is genetic control over anadromy. Natural or manmade barriers to migration (e.g., waterfalls, dams) add complexity to the relationship between sympatric life history types within a river system [9,28-31]. Barriers may promote divergence between above-barrier, resident populations and a below-barrier population containing fish of one or both life history types. One-way migration of a limited number of fish downstream over the barrier may provide a means for gene flow into the below-barrier population from the above-barrier population [32].

In this study, we 1) determine the occurrence and pathways of gene flow and degree of genetic similarity between sympatric resident and anadromous *O. mykiss* in three river systems, and 2) investigate whether resident *O. mykiss* are producing anadromous offspring in these rivers, two of which have barriers to upstream migration. 

## Methods

### Sample collection

 All necessary scientific collection permits for this study were obtained from the Washington Department of Fish and Wildlife by the National Oceanic and Atmospheric Administration (NOAA). *Oncorhynchus mykiss* is listed as a threatened species in Puget Sound under the *U*.S Endangered Species *Act* (ESA), and all ESA consultation requirements were met.

**Figure 1:**
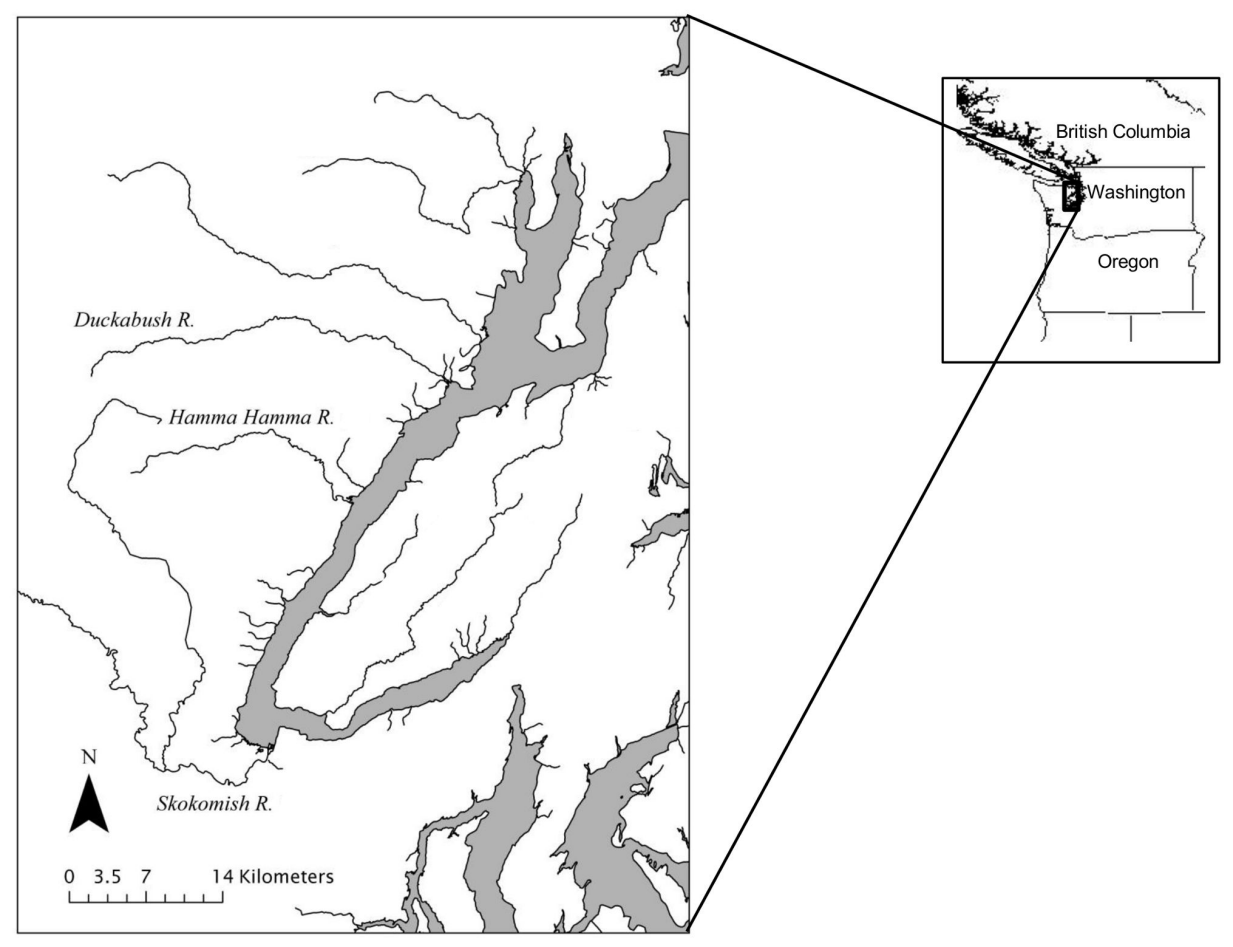
Map of the study area. The three rivers where *O. mykiss* samples were collected.

*O. mykiss* samples were collected from three rivers that flow into Hood Canal in Washington State – the Duckabush, Hamma Hamma, and South Fork Skokomish (hereafter, referred to as Skokomish) rivers (Figure 1). The majority of *O. mykiss* used for this study were sampled non-lethally by temporarily anesthetizing the fish using tricaine mesylate (MS-222), removing a small portion of fin tissue using scissors, and storing the tissue in 95% ethanol. Fish for which otolith analyses were required were killed by subjecting them to a lethal dose of MS-222. They were stored frozen at -80°C until their otoliths were removed. Resident *O. mykiss* and parr of an unknown life history type were collected via hook-and-line angling during the summer months (late July through early September, Table 1). In Hood Canal, *O. mykiss* adopting an anadromous life history (i.e., smolts) out migrate during springtime (April - June) from freshwater to marine waters, predominately at 2 years of age, and at an average size of 170 mm. Any fish greater than 200 mm remaining in the river during summertime have substantial opportunity for further growth prior to the next spring migration window, and are therefore almost certainly adopting a resident life history, and are above the minimum threshold size for maturation [5]. Based on this information, we categorized fish in our study as residents if their length was equal to or greater than 200 mm. The Duckabush River and the Hamma Hamma River each contain a natural falls (at river kilometer 12.1 and 3.8, respectively) that presents a barrier to further upstream migration by returning anadromous *O. mykiss*. Resident *O. mykiss* from these rivers were collected from both above and below the barriers. Fish that were moving downstream were also captured in the lower reaches of the rivers in rotary screw traps during April and May and were categorized as smolts, and thus were adopting an anadromous life history, if they were greater than 125 mm. This threshold size for smoltification is consistent with other populations [33]. At this size fish show silvering and loss of parr marks, development of dark margins on the fins, loosening of the scales and body elongation [34]. Ideally, we would also have collected adult anadromous *O. mykiss* from these rivers; however, that was not possible for these locations. The samples used were collected as part of an ongoing genetic monitoring study of anadromous *O. mykiss* supplementation in Hood Canal. The Duckabush and Skokomish River samples were all collected before any current supplementation efforts would have affected the samples, whereas the Hamma Hamma River samples were collected after the river had been supplemented with captively reared smolts and adults, which originated from the Hamma Hamma River, beginning in 2000 [32,35].

**Table 1:** The location, life stage (and for resident *O. mykiss*, if the fish was collected above or below a barrier to upstream migration), years sampled, and number genotyped of *O. mykiss* analyzed.

Location	Life Stage	Years Sampled	N
Duckabush River	Resident *O. mykiss* above	2006 - 2008	89
	Resident *O. mykiss* below	2006-2008, 2010, 2011	119
	Parr	2006, 2007	39
	Smolts	2007 - 2011	172
Hamma Hamma River	Resident *O. mykiss* above	2002-2005, 2008	136
	Resident *O. mykiss* below	2006-2008	85
	Parr	2006 - 2008	29
	Smolts	2007 - 2011	215
Skokomish River	Resident *O. mykiss*	2006 - 2008, 2010, 2011	111
	Parr	2006 - 2008	83
	Smolts	2006 - 2011	385

### Genotyping

We genotyped all samples for 15 microsatellite DNA loci – *Ocl1* [36], *Oke4* [37], *Oki23* [38], *Ogo4* [39], *Omy1001*, *Omy1011* [40], *Omy77* [41], *Omy325* [42], *Omy7iNRA* (K. Gharbi, University of Glasgow, Glasgow, United Kingdom, unpublished data), *Oneu14* [43], *Ots100* [44], *Ots3*, *Ots4* [45], *Ssa289* [46], *Ssa407*, and *Ssa408* [47]. Genomic DNA was isolated using Promega Wizard DNA Purification Kits (Promega Corp.), and then amplified for each locus using polymerase chain reactions (PCR). The resulting PCR products were sized using an Applied Biosystems 3100 genetic analyzer. Genotypes for each individual were determined using GeneScan and Genotyper software (Applied Biosystems Inc.).

### Within river population structure

Genetic diversity among and within rivers was measured by conducting AMOVA analyses [48] with the program Arlequin [49]. Significance was tested using 1,000 random permutations. 

**Figure 2:**
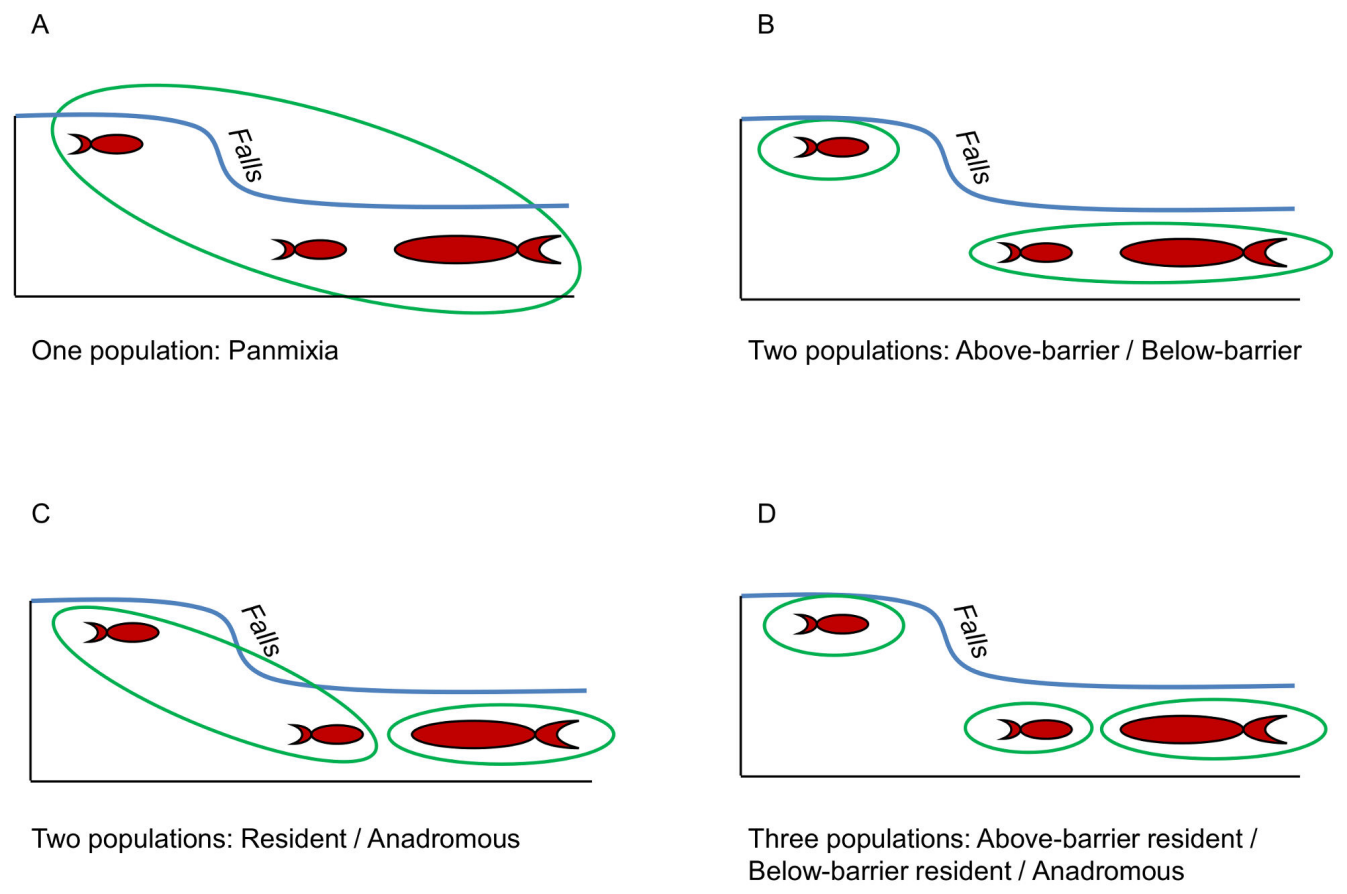
Pictorial representation of the hypothetical population structures tested for resident and anadromous *O. mykiss* samples. The small and large fish icons represent resident and anadromous *O. mykiss*, respectively, the falls represent a barrier to upstream migration, and the green ovals encompass the life-history type and location of fish considered as a single population for each scenario. For the Skokomish River, which does not contain a barrier to migration, only the one population, panmixia (A), and two population, resident / anadromous (C) scenarios were considered.

Several possible configurations of population structure within each river were tested to determine which one more closely defines the true configuration. Our samples could be sub-divided based upon life history type (resident *O. mykiss* vs. anadromous *O. mykiss*) as defined above, and for the Duckabush and Hamma Hamma rivers, based upon location above or below a barrier to upstream migration. We hypothesized that one of four different configurations of population sub-division could be present, and divided samples within each river into groups accordingly for analyses (Figure 2). We used three methods to search for the population structure configuration that showed the most differentiation among sample groups, thus representing the greatest departure from panmixia. These results allowed us to make inferences about the number of distinct *O. mykiss* populations in each river. 

First, genetic diversity among sample groups was measured by calculating pairwise *F*_*ST*_ values and corresponding 95% confidence intervals in the program FSTAT [50]. The *F*_*ST*_ values were plotted to compare their values among the different sample groups. Secondly, we performed leave-one-out tests in GENECLASS2 [51] using the method of Rannala and Mountain [52]. This test removes an individual from the sample groups and assigns it to a group as if it were a fish of unknown origin. After this is completed for every individual in the sample set, the assignments are compared to the known origin of each fish to measure the accuracy of the assignments. As gene flow among populations decreases, the percentage of correct assignments is expected to increase, as has been shown through simulations [53]. The binomial probability of the number of correct assignments observed was calculated to determine if they were greater than the number expected by chance [53]. Thirdly, we ran the program STRUCTURE [54], varying the number of populations (*K*) in each river from 1 - 5. STRUCTURE uses a Bayesian clustering analysis to infer the number of populations that exist in a sample without defining the populations a priori. We used a burn in length of 100,000 iterations followed by 200,000 iterations, using the correlated allele frequencies model. We ran 20 simulations for each value of *K* to calculate a mean log probability value (*L(K*)) for each *K* value. We then used the method of Evanno et al. [55] to calculate the value *ΔK*, the rate of change of *L*(*K*) between successive *K* values, which is a better estimator of the number of clusters in a group compared to *L*(*K*) alone. The greatest *ΔK* value was considered to be the true value of *K*.

### Parentage analysis

Parentage analyses were performed to determine whether any of the resident *O. mykiss* sampled above or below the barriers produced parr or smolts. Individuals having identical genotypes were searched for using GenAlEx [56] so we could exclude any parr-resident *O. mykiss* parentage matches that represented the same individual sampled at two different times, rather than a true parent-offspring match. CERVUS [57] was used to calculate the average number of alleles per locus and the average non-exclusion probability for the first parent. The average non-exclusion probability is the probability that a candidate parent that is not the true parent of an offspring will not be excluded as a potential parent for that offspring.

Parentage assignments of resident *O. mykiss* as candidate parents and parr and smolts as candidate offspring were made using the program CERVUS [57]. Because *O. mykiss* are iteroparous [5], the samples collected were considered as candidate parents for parr and smolt samples collected in years both before and after the resident *O. mykiss* samples were collected. We only included individuals in the parentage analyses that were genotyped for at least 12 of the 15 loci. Parental matches were accepted only if there were no allele mismatches between a candidate parent and offspring, and if the maximum likelihood confidence value for the pair was > 0.95. These were very conservative measures that resulted in fewer parent-offspring matches than if an exclusionary method or a maximum likelihood method alone was used. However, these stringent criteria serve to increase the certainty of our reported parent-offspring matches. We chose these criteria because of the potentially high number of unsampled parents in our study, as we did not have samples from any adult anadromous *O. mykiss*, and we did not know what proportion of the resident *O. mykiss* population we had sampled. Confidence values of parentage assignments are determined in CERVUS using the results of parentage simulations. For these calculations we used simulation parameters of 100,000 offspring and 500 candidate parents, and assumed that we sampled only 10% of the total number of potential parents. Although we did not have an estimate of the size of the resident *O. mykiss* populations in these rivers, once again, we believe these were very conservative parameters aimed at increasing the confidence in our results. Additional simulations were run assuming we had sampled 50% and 75% of the total number of potential parents, to assure that the parameters we had chosen were not severely biasing our results. 

### Genetic sex identification

All resident *O. mykiss* were genotyped for the locus *OmyY1* [58] to determine their genotypic sex, which then would allow us to examine if geneflow from the resident population into the anadromous population is sex-specific. We also genotyped all smolts captured in the screw traps for *OmyY1* so that the sex ratio of fish from different locations and different life histories could be compared. *OmyY1* is a male-specific marker that can be used to determine the genotypic sex of *O. mykiss* when it is amplified in combination with microsatellite loci (M. Campbell, Idaho Department of Fish and Game, Eagle, Idaho, personal communication). We combined *OmyY1* primers along with those for *Ogo4*, *Omy7*, and *Ots4* into a single PCR reaction. Genomic DNA from each resident *O. mykiss* identified as a parent was amplified and genotyped for *OmyY1* at least twice to confirm the results. The sex ratio of *O. mykiss* collected in each location was tested for deviance from an expected 1:1 ratio by calculating the observed ratio’s binomial probability. 

### Otolith analysis

Evidence for anadromous female x resident male matings was investigated first by determining maternal life history of each parr using otolith microchemistry analysis. Reliable determinations of maternal life history have been documented for these populations, including the same parr genetically analyzed in this study (see Berejikian et al. [59] for methods and parr maternity for each population). Only parr that were identified as having an anadromous *O. mykiss* mother from the otolith analyses were then analyzed to determine their paternity by the parentage analysis described above. In this manner, we were able to conclude if anadromous females were only spawning with anadromous males, or if some resident males were also spawning with the anadromous females. Due to the need to lethally sample fish to obtain their otoliths and the conservation concerns pertaining to these populations, we did not sample any smolts for otolith analyses.

## Results

### Genotyping results

Samples from all three rivers were highly polymorphic as the mean number of alleles per locus was 16.0 for the Duckabush River, 16.4 for the Hamma Hamma River, and 14.9 for the Skokomish River. The average non-exclusion probability of the first parent for all populations was < 0.00009. Four individuals (one resident *O. mykiss*, two smolts, one parr) were found to have genotypes matching other individuals, and were subsequently dropped from any further analyses, as they were most likely individuals that had been sampled at two different times.

### Within river population structure

The AMOVA analyses revealed that more variation existed among the different rivers (3.3%), then among differing samples within each river (2.6%) . Both of these values are significantly greater than zero (*P* < 0.001). 

**Figure 3:**
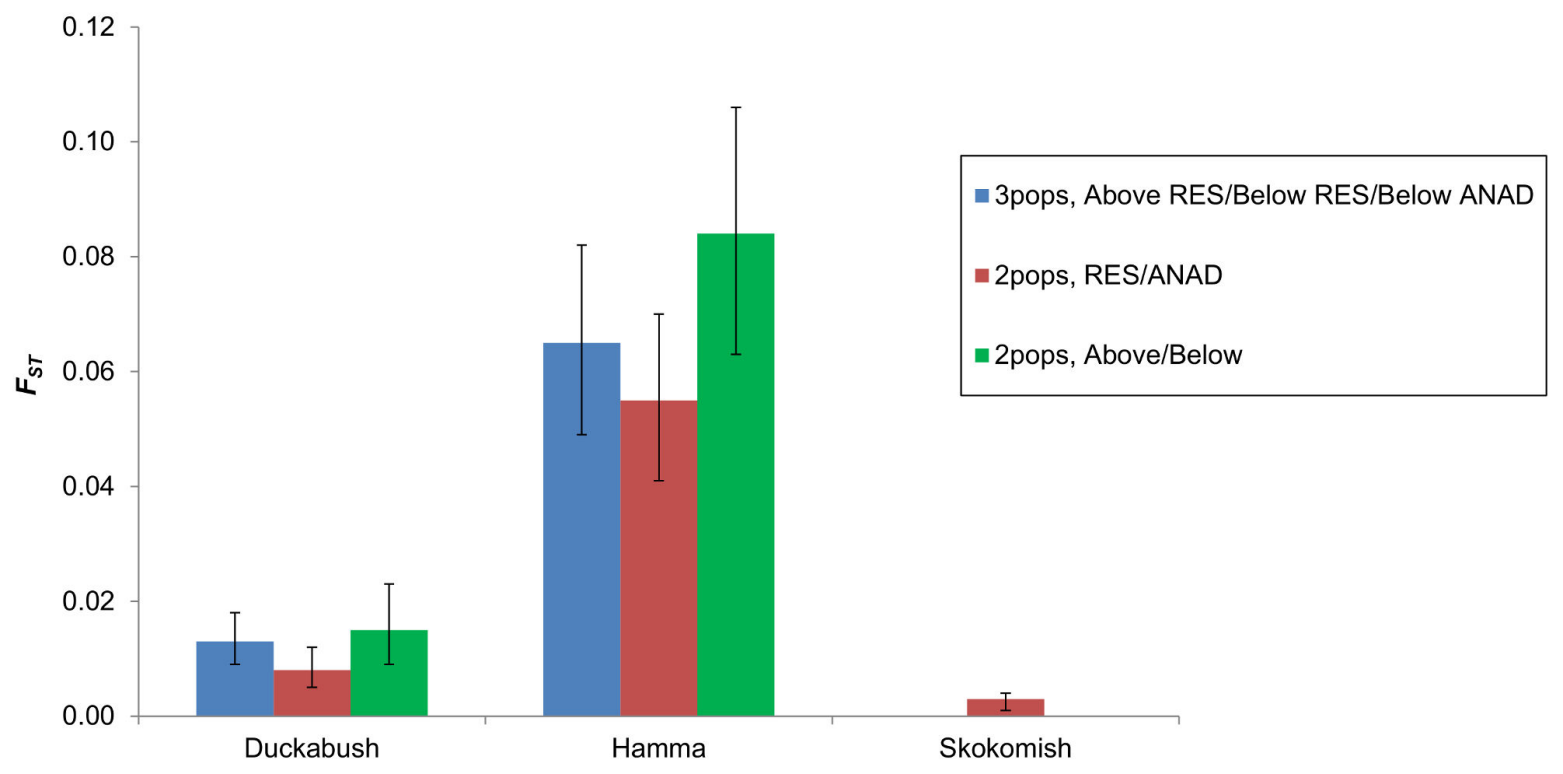
Pairwise *F*_*ST*_ estimates for resident (RES) and anadromous (ANAD) *O. mykiss* sampled from three rivers. Samples are grouped in various population structure configurations depending on their location above or below a barrier to upstream migration, and life history type.

The pairwise *F*_*ST*_ values showed some differences among the different population configurations within each river, but none of them were significantly different from one another as all of the 95% confidence intervals overlapped (Figure 3). The two population, above / below configuration had the greatest *F*_*ST*_ values for both the Duckabush and Hamma Hamma river samples. For the Skokomish River, which lacks a barrier to migration, the two population, resident / anadromous *O. mykiss* configuration had a very low *F*_*ST*_ value (0.003) that was just barely above zero (lower 95% confidence interval = 0.001). This value is also lower than similar comparisons (below resident *O. mykiss* vs. below anadromous *O. mykiss*; not shown on figure) in the Duckabush River (*F*_*ST*_ = 0.005) and the Hamma Hamma River (*F*_*ST*_ = 0.016).

**Figure 4:**
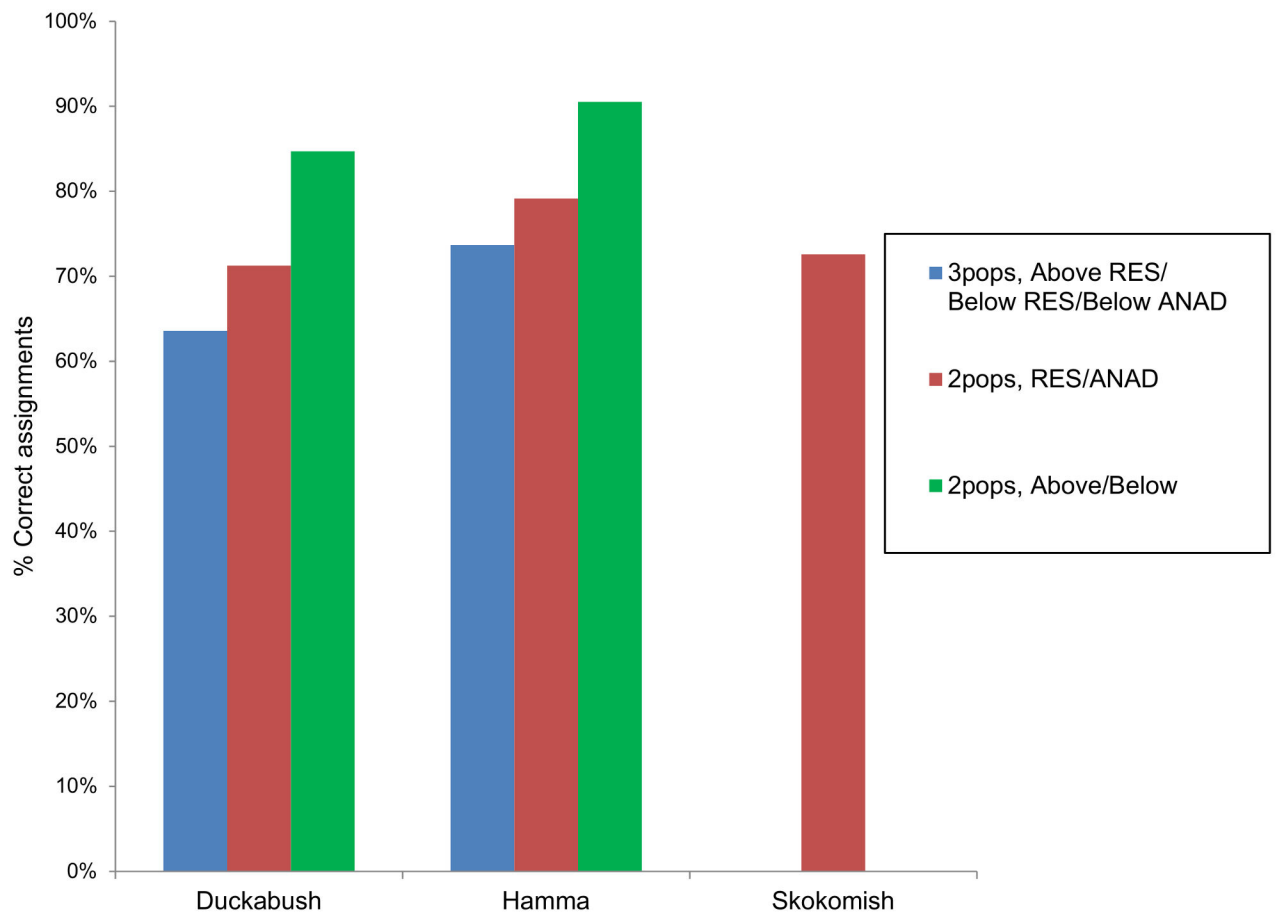
Leave-one-out assignment tests for resident (RES) and anadromous (ANAD) *O. mykiss* sampled from three rivers. Samples are tested for the percent of correct assignments to population of origin given different population structure configurations depending on sample’s location above or below a barrier to upstream migration, and life history type.

Results from the leave-one-out tests suggest that for all population structure configurations tested, the number of correct assignments was significantly greater than random expectations (*P* < 0.001; Figure 4). For the Duckabush and Hamma Hamma rivers, the two populations, above / below configuration had the greatest number of correct assignments, with 84.7% and 90.5% correct, respectively. The only configuration tested for the Skokomish River (two populations, resident *O. mykiss* / anadromous *O. mykiss*), had a correct assignment value of only 72.6%.

**Figure 5:**
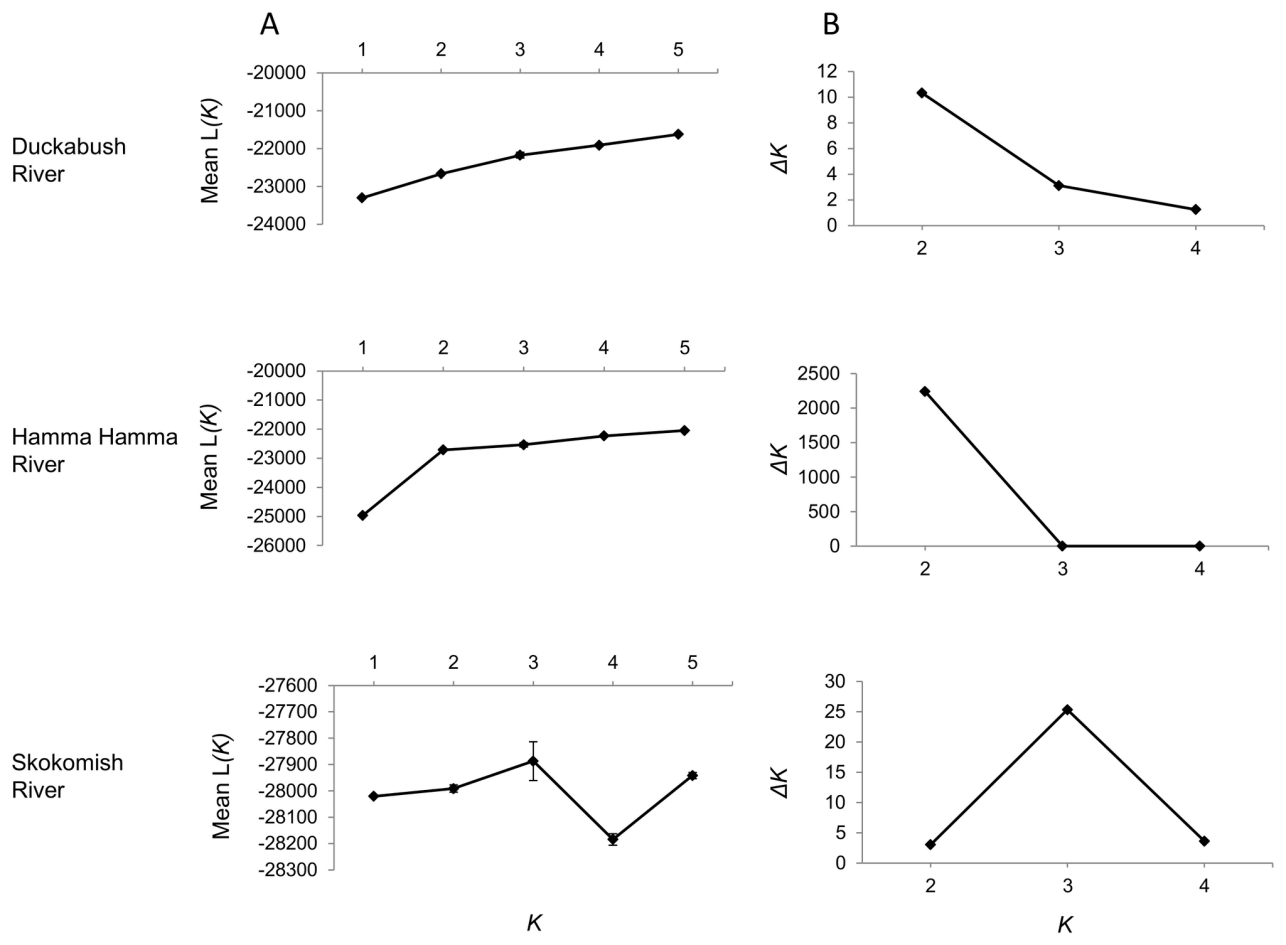
Most likely number of *O. mykiss* populations (*K*) in three rivers determined from STRUCTURE analyses. The most likely value of *K* is the one with the greatest rate of change (*ΔK*) between successive L(*K*) values.

The STRUCTURE analyses found that for the Duckabush and Hamma Hamma rivers, the greatest *ΔK* was obtained when *K* = 2 (Figure 5). The values of *ΔK* for the Hamma Hamma River are considerably greater than those of the other rivers, suggesting stronger differentiation between the above and below Hamma Hamma River populations than those in the Duckabush River. Initially, it appears that the highest *ΔK* for the Skokomish River is when *K* = 3, a value not even considered in our population configurations. However, *ΔK* cannot find the best *K* if the true value of *K* = 1 and will return illogical results [55]. In addition, the *L*(*K*) values for the Skokomish River do not follow the typical pattern of continually increasing values, most likely due to the fact that substantial gene flow between populations can reduce the reliability of estimating the true value of *K* from STRUCTURE results [53]. Thus, we did not consider the STRUCTURE estimate to be accurate, as our other two methods provided strong evidence that *O. mykiss* in the Skokomish River consist of a single panmictic population.

### Parentage results

A total of 19 (2.5%) of the 772 smolts examined were identified as offspring of resident *O. mykiss* (Table 2). It is important to note that these proportions should be considered the minimum proportion of smolts that had a resident parent, as we most likely did not sample every resident in each stream. Thus, other smolts could have been the offspring of unsampled residents. Parentage results using simulation data where we assumed we had sampled 50% or 75% or the total number of potential parents did identify a small number of additional offspring-parent matches (N = 1 & 1, Duckabush River; N = 3 & 6, Hamma Hamma River; N = 1 & 3, Skokomish River), which showed that we were not severely biasing our results with our chosen parameters. Of the resident *O. mykiss* identified as parents of smolts, 17 (89.5%) were males and only 2 (10.5%) were females. Three male resident *O. mykiss* were identified as the parent of two smolts each, and one male was identified as the parent of both a smolt and a parr. All other parents identified matched just one smolt or parr each. No smolts could be identified as coming from a resident x resident mating, as none of them were assigned to both a male and female resident *O. mykiss*. Once again, this result does not preclude that possibility, as it is highly likely that there were resident *O. mykiss* present in these populations that we did not sample. None of the resident *O. mykiss* that were collected above the barrier to upstream migration in the Hamma Hamma River were identified as parents of any smolts. However, one smolt from the Duckabush River was identified as an offspring of a female resident *O. mykiss* collected above the barrier.

**Table 2:** The number and percentage of smolts in samples from each river that had a resident *O. mykiss* parent.

River	Male Parent	Female Parent	Percent of Sample
Duckabush	9	2	6.4%
Hamma Hamma	6	0	2.8%
Skokomish	2	0	0.5%

Four of the 150 parr examined were matched to a resident *O. mykiss* parent (2 in the Duckabush River; 1 each in the Hamma Hamma and Skokomish rivers). All of these parents were identified as males by the genotypic sex analysis. Because we know from otolith analyses that all of these parr had an anadromous maternal origin, they must have been produced by a male resident *O. mykiss* spawning with a female anadromous *O. mykiss*. 

### Sex ratio

The sex ratio of all populations of residents and smolts deviated significantly from the expected 1:1 ratio (Table 3). For the above-barrier resident populations in the Duckabush and Hamma Hamma rivers, we found significantly more females than males, as was also the case for outmigrating smolts, caught in the screw traps, from each of the three rivers. Conversely, in the below-barrier resident populations in the Duckabush and Hamma Hamma rivers, and in the Skokomish River resident *O. mykiss* population, males made up a significantly greater proportion of each population.

**Table 3:** Total number of female and male *O. mykiss* found in each sampling location, the female : male ratio, and the probability that the observed sex ratio is 1:1.

Location	*N* total	*N* females	*N* males	Ratio	*P*
Duckabush River, Above-barrier residents	89	60	29	2.1	0.001
Duckabush River, Below-barrier residents	119	44	75	0.6	0.003
Duckabush River, Screw trap smolts	135	94	41	2.3	0.000
Hamma Hamma River, Above-barrier residents	136	93	43	2.2	0.000
Hamma Hamma River, Below-barrier residents	85	29	56	0.5	0.002
Hamma Hamma River, Screw trap smolts	208	130	78	1.7	0.000
Skokomish River, In river residents	111	30	81	0.4	0.000
Skokomish River, Screw trap smolts	291	169	122	1.4	0.003

## Discussion

The population structure of the *O. mykiss* in these rivers appears to be influenced more by the presence of a barrier to upstream migration than by the life history type of the fish. Our results show that in areas within a river where resident and anadromous *O. mykiss* are able to mix freely, there is less genetic differentiation between the life history types than there is between fish sampled above and below the barrier (i.e., Figure 2B). However, there does appear to be enough gene flow between fish above and below the barriers to prevent complete reproductive isolation. Although the barriers present in the Duckabush and Hamma Hamma rivers presumably block gene flow from below-barrier *O. mykiss* into the above-barrier population, gene flow is not necessarily blocked in the opposite direction. Results from previous studies have implied that resident *O. mykiss* are capable of surviving a descent over waterfalls [18,31], including those in the Hamma Hamma River [32]. Thus, the potential does exist for gene flow between the above-barrier and below-barrier populations, albeit in a single direction. Our results suggest that gene flow between the above-barrier and below-barrier populations has been more substantial in the Duckabush River, as evidenced by its much lower AMOVA values between the above-barrier and below-barrier groups compared to the Hamma Hamma River. However, past stocking practices of hatchery raised *O. mykiss* has also likely affected the population structure in the Hamma Hamma River. Non-native resident *O. mykiss*, derived from the McCloud River, California, were regularly stocked into the Hamma Hamma River, above its barrier to upstream migration, from the mid-1970s through 1996 [59]. If the introduced fish were reproductively successful and introgressed into the gene pool of the native population, an increased level of genetic differentiation between life history types and locations would have occurred, as non-native genes from the hatchery stock were introduced only into the above-barrier resident *O. mykiss* population. This is evident when our results from the Hamma Hamma River are compared to the other populations, which have not received any reported releases of non-native *O. mykiss*. The Hamma Hamma River samples had much greater pairwise F_ST_ values for all of the population configurations tested, and the *ΔK* values from the STRUCTURE analyses were several magnitudes greater than they were for either the Duckabush or Skokomish River samples. We would expect that over time, the non-native genes introduced above the barrier will become introgressed into the below-barrier population, but the speed at which this occurs depends upon the level of gene flow between locations via fish migrating downstream over the barrier, and the reproductive success of the hatchery stock relative to the native fish.

The sex ratios we observed in the above-barrier resident *O. mykiss* samples suggest that migration over the barriers may be sex biased. The above-barrier samples were highly female-skewed, whereas the below-barrier samples were male-skewed, indicating that males migrate downstream over the barriers at a greater frequency then females. This is especially important given our finding that gene flow from the resident populations into the anadromous populations occurs primarily via resident males spawning with anadromous females. Sex-biased migration of resident males over the barriers into lower reaches of the river would increase the number of resident males in the steelhead spawning areas, and thus, increase the opportunities for resident male and anadromous female matings. Resident *O. mykiss* are known to be highly migratory in freshwater systems [11,60], but in contrast to our results, Olsen et al. [11] found no evidence of sex-biased dispersal. However, in that study, dispersal was measured between locations within a river that were not separated by a barrier to migration, as in our study. Sex-specific density dependent factors above the barrier, such as competition among males for mates, could drive some males to venture over the barrier in search of areas with less competition. There could also be other explanations for our results, such as higher mortality in males in the above-barrier locations accounting for the female-skewed ratio in those locations, or precocious male maturation in anadromous *O. mykiss*, accounting for the male-skewed ratios in below-barrier populations (discussed below).

The *F*_*ST*_ values we calculated were significantly greater than zero when considering the two populations (resident and anadromous), indicating some degree of reproductive isolation between life history types, especially in the Hamma Hamma River. The assignment tests for the configuration that considered both a resident and anadromous population were significantly more accurate than what would be expected by chance if only a single panmictic population existed. Reproductive isolation between life history types may be promoted by size assortative mating, evident in some salmonids [61,62], or partial spatial [7] or temporal [12] isolation in spawning. However, even a low mating frequency between life history types could result in substantial gene flow between them, thus partially counteracting divergence caused by genetic drift or selection [63].

Our results provide direct confirmation through paternity analyses that resident *O. mykiss* produce offspring that become anadromous. This was especially true in the Duckabush River, where we found the greatest proportion of smolts (6.4%) that had a resident *O. mykiss* as one of their parents. This could be related to the relatively low numbers of returning anadromous *O. mykiss* that have been observed there. For example, the annual average number of anadromous *O. mykiss* redds observed from 2008-2010 was 11.7 for the Duckabush River, 64.3 for the Hamma Hamma River, and 249.7 for the Skokomish River [64]. If there are few anadromous male *O. mykiss* on the spawning grounds, some resident males may be spawning with anadromous females with little to no competition from anadromous males. Our results suggest this is occurring, as we found four parr that were the offspring of matings between male resident and female anadromous *O. mykiss*. This could create what Araki et al. [24] called “genetic compensation between life history forms,” and speculated that it could be a means of stabilizing the effective size of an *O. mykiss* population during times of low anadromous *O. mykiss* abundance. A study by Berejikian et al. [59] found much higher proportions of below-barrier, *O. mykiss* parr with resident mothers in the Duckabush (42.0%) and Hamma Hamma (58.7%) rivers compared to the Skokomish River (5.6%). They attributed the greater percentage of offspring with resident *O. mykiss* mothers in the Duckabush and Hamma Hamma rivers to the presence of large-scale habitat features and the presence of an above-barrier resident population for each of those rivers. This suggests that resident *O. mykiss* comprise a significant proportion of the *O. mykiss* populations in those two rivers, providing ample opportunities for matings between resident males and anadromous females.

Further gene flow between life history types in these rivers may be occurring due to precocious male maturation of anadromous *O. mykiss* offspring. Precocious male maturation occurs when male offspring of anadromous *O. mykiss* mature in freshwater without ever migrating to salt water, essentially adopting a resident *O. mykiss* life history. As discussed earlier, the male-skewed sex ratio in the below-barrier resident *O. mykiss* locations may be at least partially attributable to sex-biased migration over the barrier. However, no barrier exists in the South Fork Skokomish River, so the male-skewed sex ratio we observed there is likely due to precocious male maturation. This can also be clearly seen in the sex ratios of the smolt samples captured in screw traps (Table 3). Those results show that for all three rivers, significantly more females are outmigrating to marine waters then are males. McMillan et al. [12] also found a male-dominated population of wild resident *O. mykiss* in a Washington state river that did not have a barrier to migration, and Rundio et al. [65] found a male-skewed sex ratio in a California resident *O. mykiss* population that they attributed to differential rates of anadromy between sexes. We presume that precocious males would be more likely to spawn with resident *O. mykiss* due to their similar life history characteristics, thus providing a means of gene flow from the anadromous *O. mykiss* population into the resident *O. mykiss* population.

Conservation of life history diversity is important to the long term persistence of a population, as a population with life history diversity may be able to better withstand catastrophic events that might cause the extinction of a singular life history type [1,63]. Our evidence of significant gene flow between *O. mykiss* life history types is important to conservation and management issues related to this species [66]. Two of the more important issues considered are 1) in which rivers should sympatric resident and anadromous *O. mykiss* be managed as a single population versus multiple populations, and 2) do resident *O. mykiss* represent a repository of genes for a given river that can be used to restore the anadromous life history type if it has been lost (due to migration barriers, for example; [29,67])? Our results add to the growing body of evidence that shows that in many rivers gene flow does readily occur between resident and anadromous *O. mykiss* life history types. The sympatric resident and anadromous *O. mykiss* present in each of the locations we sampled do not constitute reproductively isolated populations, and appear to have a low level of gene flow between them. This is important because when a particular life history type is not entirely under genetic control, the loss of one life history type would not necessarily be permanent if that type can arise from the surviving type [68].

As for the second question, the barriers in our study area created more reproductive isolation than life history type did, but there was still enough gene flow present to prevent complete reproductive isolation. In the Duckabush River, we identified a smolt as having a mother who originated from above the anadromous barrier in that system, indicating that for that location the resident *O. mykiss* isolated above the barrier are capable of producing offspring that will adopt an anadromous life history. Our results, added to the results of previous studies [7,19-21,25,69], clearly show that resident *O. mykiss* can produce offspring that become anadromous, thereby suggesting that resident *O. mykiss* populations may represent a repository of genes for an anadromous population. However, the rate at which residents produce anadromous migrants, and the survival of those migrants, will need to be determined to help us understand the net contribution that resident *O. mykiss* can make to anadromous *O. mykiss* productivity.
